# Turning a lost reef ecosystem into a national restoration program

**DOI:** 10.1111/cobi.13958

**Published:** 2022-08-07

**Authors:** Dominic McAfee, Ian M. McLeod, Heidi K. Alleway, Melanie J. Bishop, Simon Branigan, Sean D. Connell, Craig Copeland, Christine M. Crawford, Ben K. Diggles, James A. Fitzsimons, Ben L. Gilby, Paul Hamer, Boze Hancock, Robert Pearce, Kylie Russell, Chris L. Gillies

**Affiliations:** ^1^ School of Biological Sciences The University of Adelaide Adelaide South Australia Australia; ^2^ Environment Institute The University of Adelaide Adelaide South Australia Australia; ^3^ TropWATER, Centre for Tropical Water and Aquatic Ecosystem Research James Cook University Townsville Queensland Australia; ^4^ The University of Adelaide Adelaide South Australia Australia; ^5^ Provide Food and Water The Nature Conservancy Arlington Virginia USA; ^6^ School of Natural Sciences Macquarie University Sydney New South Wales Australia; ^7^ The Nature Conservancy Australia Carlton Victoria Australia; ^8^ OzFish Unlimited Ballina New South Wales Australia; ^9^ Institute of Marine and Antarctic Studies University of Tasmania Hobart Tasmania Australia; ^10^ DigsFish Services Pty Ltd Brisbane Queensland Australia; ^11^ School of Life and Environmental Sciences Deakin University Melbourne Victoria Australia; ^12^ School of Science and Engineering University of the Sunshine Coast Sunshine Coast Queensland Australia; ^13^ Victorian Fisheries Authority Melbourne Victoria Australia; ^14^ The Nature Conservancy, c/o Graduate School of Oceanography University of Rhode Island Kingston Rhode Island USA; ^15^ Albert Park Yachting and Angling Club Albert Park Victoria Australia; ^16^ NSW Department of Primary Industries Taylors Beach New South Wales Australia

**Keywords:** ecosystem restoration, environmental management, marine policy, oyster reef, shellfish habitat, arrecife de ostras, gestión ambiental, hábitat de conchas, política marina, restauración de ecosistemas

## Abstract

Achieving a sustainable socioecological future now requires large‐scale environmental repair across legislative borders. Yet, enabling large‐scale conservation is complicated by policy‐making processes that are disconnected from socioeconomic interests, multiple sources of knowledge, and differing applications of policy. We considered how a multidisciplinary approach to marine habitat restoration generated the scientific evidence base, community support, and funding needed to begin the restoration of a forgotten, functionally extinct shellfish reef ecosystem. The key actors came together as a multidisciplinary community of researchers, conservation practitioners, recreational fisher communities, and government bodies that collaborated across sectors to rediscover Australia's lost shellfish reefs and communicate the value of its restoration. Actions undertaken to build a case for large‐scale marine restoration included synthesizing current knowledge on Australian shellfish reefs and their historical decline, using this history to tell a compelling story to spark public and political interest, integrating restoration into government policy, and rallying local support through community engagement. Clearly articulating the social, economic, and environmental business case for restoration led to state and national funding for reef restoration to meet diverse sustainability goals (e.g., enhanced biodiversity and fisheries productivity) and socioeconomic goals (e.g., job creation and recreational opportunities). A key lesson learned was the importance of aligning project goals with public and industry interests so that projects could address multiple political obligations. This process culminated in Australia's largest marine restoration initiative and shows that solutions for large‐scale ecosystem repair can rapidly occur when socially valued science acts on political opportunities.

## Abstract 2

*Article impact statement*: Integrating sociopolitical goals and community stewardship into restoration planning builds support to enable large‐scale restoration.

## Abstract (es)

Transformación de un Ecosistema Arrecifal Perdido en un Programa Nacional de Restauración

**Resumen:**

Actualmente se requiere una reparación ambiental a gran escala que atraviese fronteras legislativas para lograr un futuro socio‐ecológico sustentable. Aun así, habilitar la conservación a gran escala es complicado debido a los procesos de elaboración de políticas que están desconectadas de los intereses socio‐económicos, las múltiples fuentes de conocimiento y las diferentes aplicaciones de las políticas. Consideramos cómo una estrategia multidisciplinaria para la restauración de hábitats marinos generó una base de evidencia científica, apoyo comunitario y el financiamiento necesario para así iniciar la restauración de un ecosistema arrecifal de conchas funcionalmente extinto. Los actores clave formaron una comunidad multidisciplinaria de investigadores, practicantes de la conservación, comunidades de pescadores recreativos y órganos gubernamentales que colaboró con varios sectores para redescubrir los arrecifes perdidos de Australia y comunicó el valor de su restauración. Las acciones realizadas para armar el caso para la restauración marina a gran escala incluyeron la síntesis del conocimiento actual sobre los arrecifes de conchas en Australia y su declinación histórica, el uso de esta historia para contar una narración convincente que active el interés público y político, la integración de la restauración a la política gubernamental y la movilización del apoyo local por medio de la participación comunitaria. Claramente, la articulación del caso del negocio social, económico y ambiental para la restauración llevó al financiamiento estatal y nacional para la restauración arrecifal a cumplir diversos objetivos socio‐económicos (p. ej.: creación de empleos, oportunidades recreativas) y de restauración (p. ej.: una productividad realzada de la biodiversidad y las pesquerías). Una lección clave que aprendimos fue lo importante que es alinear los objetivos del proyecto con los intereses públicos y de la industria, de tal manera que los proyectos aborden las múltiples obligaciones políticas. Este proceso culminó con la iniciativa de restauración marina más grande en Australia y demuestra que las soluciones para la reparación de los ecosistemas a gran escala pueden ocurrir rápidamente cuando la ciencia con valor social actúa sobre las oportunidades políticas.

## INTRODUCTION

Marine ecosystem restoration could play a foundational role in meeting humanity's goals (e.g., UN's Sustainable Development Goal 14: Life Below Water) for a sustainable socioecological future (Duarte et al., 2020). Most biogenic marine ecosystems are degraded worldwide (e.g., seagrass, kelp forests, coral reefs, and shellfish reefs), yet the foundations for recovery often remain available to restore ecosystems that have long lost their ecological function and productivity (Lotze et al., 2006). The active restoration of marine ecosystems in current management settings has only recently been adopted relative to terrestrial restoration and has been dominated by relatively small‐scale activities compared with the scale of loss (Bellwood et al., 2019). Reasons for the slow adoption of large‐scale projects include a lack of social and political confidence in the benefits of marine restoration––stemming from perceptions of low success rates, high implementation costs, and low economic returns––and the risks of working in the marine environment (Bayraktarov et al., 2016; Saunders et al., 2020; Stewart‐Sinclair et al., 2020). This perception of high risk and low returns is compounded by the limited duration of monitoring and evaluation for most marine restorations and a general lack of reporting on their socioeconomic benefits (Bayraktarov et al., 2016). Yet, large‐scale marine restoration is gaining momentum and some “bright spots” have demonstrated large‐scale recoveries (Saunders et al., 2020) and returns‐on‐investment that increase with project size (Hernández et al., 2018). These successes are building optimism that marine restoration can play a central role in a more sustainable future (Duarte et al, 2020). To scale up marine restoration efforts, multidisciplinary teams are needed to work together to address the social, ecological, and economic challenges that currently prevent large‐scale adoption (Waltham et al., 2020).

**FIGURE 1:**
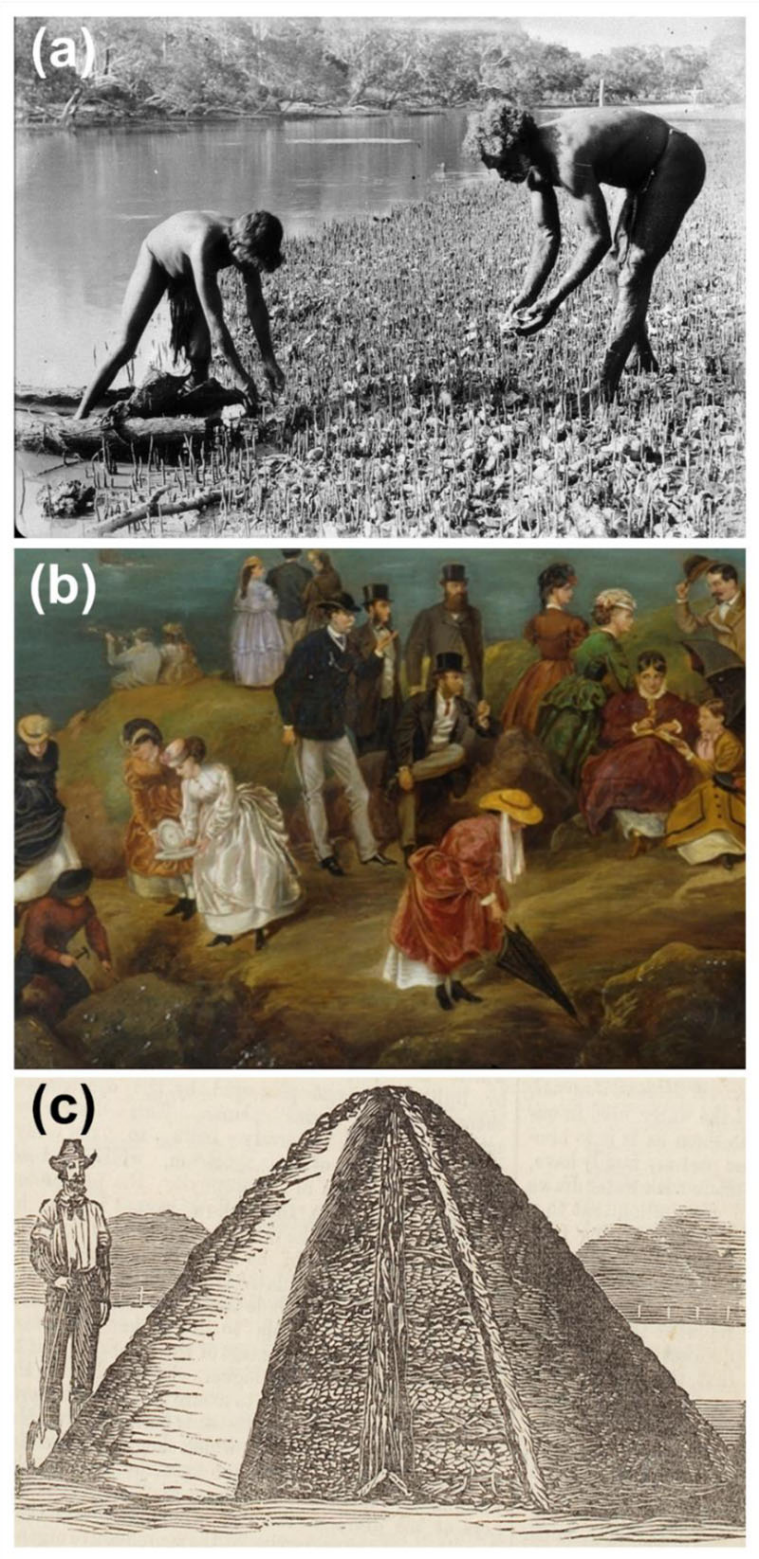
Historical images showing the past social and economic value of oysters in Australia: (a) Indigenous people harvested and managed oyster reefs over millennia (oyster harvesting in Port Macquarie, NSW, 1905 [NSW State Library]), (b) European settlers in Australia collected oysters at oystering parties (extract from Montagu Scott's 1870s painting *A Day's Picnic on Clark Island, Sydney Harbour* [NSW State Library]) and extracted oyster reefs as one of colonial Australia's first large‐scale fisheries, and (c) an 1874 newspaper engraving showing how piles of oyster shell were burned to make lime for fertilizers and cement production (*Australian Town and Country Journal*)

Shellfish reefs, primarily those formed by oysters and mussels, have supported coastal societies with food and material resources across the world for millennia. Today, however, they are among the most degraded marine ecosystems worldwide with 85% of oyster reefs lost globally (Beck et al., 2011). In Australia, for example, shellfish reefs provided protein and cultural resources for Indigenous Australians for thousands of years (Attenbrow, 2012; Reeder‐Myers et al., 2022), and were of high socioeconomic value as one of colonial Australia's first large‐scale fisheries (Nell, 2001; Schrobback et al., 2014) (Figure 1). Shellfish reefs formed the primary biogenic habitat in the bays and estuaries of over 7000 km of Australian coastline when European settlers arrived (Gillies et al., 2018; McAfee & Connell, 2021), but the 19th‐century oyster fishery and lime industry largely eradicated the ecosystem within 100 years (Schrobback et al., 2014; Alleway & Connell, 2015), with losses compounded by declining water quality, disease, and siltation from catchment development (Diggles, 2013). Today, Australia's shellfish reefs are considered functionally extinct (Beck et al., 2011); less than 1% of Australian flat oyster (*Ostrea angasi*) and 8% of Sydney rock oyster (*Saccostrea glomerata*) reefs remain (Gillies et al., 2018). Australia's shellfish reefs were lost from the collective memory of successive generations (Alleway & Connell, 2015), and few people now know these reefs ever existed.

Although Australia's shellfish reefs are nearly extinct (Gillies et al., 2020), their restoration offers tremendous potential to revive the ecological services they once provided society (McAfee et al., 2020a). For example, the restoration of 5199 ha of shellfish reef in the United States has occurred following an extensive historical loss of shellfish ecosystems (Hernández et al., 2018). Success stories from large‐scale restorations in the United States have demonstrated rapid recoveries of shellfish populations and their ecological services (Schulte et al., 2009) and generated considerable public engagement; tens of thousands of volunteers have contributed to U.S.‐based shellfish restorations (Schrack et al., 2012). Such examples are encouraging for the adoption of large‐scale marine restoration in Australia, where 85% of the population lives within 50 km of the coast and many local communities engage in coastal stewardship (Gillies et al., 2015). Additionally, Australia's well‐established shellfish aquaculture industry and the politically influential recreational fishing community that regularly supports habitat conservation provide political and funding opportunities to leverage local support and technical expertise for large‐scale restoration. These underlying conditions, discussed below, may present large‐scale restoration as an attractive management solution for achieving multiple socioeconomic and environmental goals, provided science and community engagement informs decision‐making (e.g., McAfee et al., 2021a).

We considered how a multidisciplinary approach to marine habitat restoration has established a new paradigm in the awareness, science, and recovery of Australian shellfish reef ecosystems. We characterized the key enablers and summarized actions and research that came together to bring this ecosystem from the relatively unknown into the national spotlight as the focus of Australia's largest marine restoration program, Reef Builder. We also examined how initial sociopolitical inertia and logistical hurdles to establishing an ecosystem‐wide marine restoration program were overcome, which may help others meet the global challenge of scaling‐up the recovery of marine ecosystems.

## FIRST STEPS TOWARD A RESTORATION BLUEPRINT

Indigenous Australians sustainably managed and harvested oyster reefs over millennia with ancestral practices, such as building artificial reefs with stone and shell to replenish oyster populations (Frawley, 2017; Reeder‐Myers et al., 2022). This sustainable use ended with the dispossession of Indigenous Australians of their lands and waters. The overharvesting of oyster reefs by colonial industries led to widespread ecological collapse and the subsequent adoption of oyster aquaculture (beginning ∼1870) to sustain the colonial fishery (Nell, 2001). Since then, sustained research and environmental monitoring by the aquaculture industry has influenced government management of coastal water quality (Schrobback et al., 2014) and generated considerable knowledge that would eventually underpin contemporary restoration efforts (discussed below).

A decade prior to progress being made on shellfish reef restoration in Australia, increased planning and community engagement on marine resource management was laying foundations for more coordinated marine conservation (Appendix S1). For example, government bodies dedicated to repairing aquatic habitat were created (e.g., New South Wales's [NSW] Aquatic Habitat Rehabilitation Unit) and established funding programs to restore fish habitat in partnership with end users (e.g., recreational fishers). These small‐scale projects, often led by fisher and community groups with government support (e.g., recreational fishing trusts that convert fishing licenses into a project funding [Appendix S1]), helped build awareness and confidence in habitat restoration as important government‐supported work with considerable community engagement. Later, in 2012, at a time when interest in local‐scale losses of shellfish reefs was gaining interest (Hamer et al., 2013; Diggles et al., 2013), the global environmental organization, The Nature Conservancy (TNC), convened a workshop with scientists, restoration practitioners, and policymakers to inform TNC's conservation priorities for temperate marine Australia. The consensus was to focus on in‐water habitat restoration in bays and estuaries (Fitzsimons et al, 2015). This workshop helped TNC secure philanthropic funding to develop a dedicated marine program––TNC's Great Southern Seascapes––and established working partnerships and leveraged opportunities with government bodies that would facilitate future restoration activities.

Initial steps toward realizing a dedicated restoration program for Australian shellfish reefs began in 2015. A seminal marine restoration workshop brought together 23 researchers, conservation practitioners, and government representatives to discuss opportunities for large‐scale (ecosystem‐wide) marine restoration with an emphasis on shellfish reefs. Five key priority areas were identified to galvanize public support and investment for restoration (Gillies et al., 2015): creating awareness of the ecological loss and building confidence that the ecosystem can be restored (priority 1); building a business case for repair (priority 2); developing policy frameworks for marine restoration (priority 3); developing skills and experience in restoration practitioners (priority 4); and learning from other restoration initiatives (priority 5).

Many key actors and actions were identified as critical to addressing these priority areas. The major recommendation from the workshop was that a multidisciplinary community of national researchers, conservationists, and government bodies should work collectively to rediscover Australia's lost shellfish reefs and communicate the value of their restoration in the broadest sense: an opportunity for society to create a positive environmental legacy and reestablish a connection to a forgotten ecosystem. To achieve this, four early steps propelled action. First, current knowledge on shellfish reefs and their historical decline was synthesized to identify knowledge gaps, a trajectory for recovery, and a compelling story that could spark public and political interest (priority area 1). Second, the social, economic, and environmental business case for restoration (priority area 2) was clearly articulated to rationalize research efforts, engage communities, secure investment, and guide construction, much like any infrastructure project. To do this, a lead organization that could work across science, policy, and practice was important to facilitate and coordinate actions. Third, an agreement was reached to establish a national, multidisciplinary network of scientists, practitioners, and coastal managers to facilitate cross‐sector ties and better coordination across the broad range of actors (priority area 4). Fourth, the experience and success of restoration campaigns abroad (primarily the United States) and local expertise (i.e., aquaculture industry) were leveraged to fill knowledge gaps until local information could be acquired (priority area 5).

These components were highlighted as key ingredients to further Australian shellfish reef restoration. Early progress was coordinated by TNC, which brought a wealth of experience from their U.S.‐based shellfish restorations (60 reef restorations by 2015), where they regularly partner with government agencies (e.g., National Oceanic and Atmospheric Administration) and scientific communities to connect research to policymaking (Schrack et al., 2012; Fitzsimons et al., 2015).

## SYNTHESIZING CURRENT KNOWLEDGE AND ECOLOGICAL HISTORY

Ecological history legitimizes the case for intervention by providing concrete data on how environments and an ecosystem's potential productivity have changed (McAfee et al., 2020b). Although rates of environmental change mean that ecological history alone cannot inform benchmarks for restoration targets, ecological history plays many roles for different stakeholders (zu Ermgassen et al., 2016a). For policymakers, historical baselines provide a conceptual model for ecosystem repair, without which debate on the reality of environmental change can delegitimize investment in restoration (Jackson & Hobbs, 2009). For the public, ecological history provides engaging storylines and imagery (historical photos and maps) on environmental change that can even engage those disengaged with the environment (Kittinger et al., 2015). This is particularly important for building a common understanding for public support; an engaging historical narrative provides a nexus for aligning public interests and policy goals, creating legitimacy for restoration as government policy (McAfee et al., 2020b).

As identified in the restoration workshop, the first priority research to support the case for restoration was reviewing and synthesizing collective knowledge on the past and present state of Australia's shellfish ecosystems (priority area 1). For rapid dissemination and momentum building, this synthesis was first published as a report presenting the collective knowledge on shellfish reef habitats nationwide (Appendix S2). This first national report provided an A‐to‐Z on shellfish reefs: what they are, their historical extent and present‐day condition, their restoration potential, and the socioeconomic benefits of their conservation. This report was followed by an in‐depth national analysis of their past, present, and potential future states (Gillies et al., 2018) and an ecological risk assessment in which the IUCN Red List of Ecosystems framework was used to confirm the critically endangered status of shellfish reef ecosystems formed by the main reef‐building oysters, *O. angasi* and *S. glomerata* (Gillies et al., 2020).

State‐by‐state reviews of the history of shellfish reefs detailed their former extent and how they were valued, exploited, and affected by colonial society (Appendix S2 & Figure 1). Insight into shellfish use by Indigenous Australians was primarily inferred from archaeological midden research, whereas contemporary Indigenous perspectives were gathered through Indigenous knowledge workshops (McLeod et al., 2018). This synthesis of historical distribution and sociocultural use of shellfish reefs accessed diverse scientific and gray literature, such as 19th‐century colonial media (Thurstan et al., 2020), to build on earlier historical research (e.g., Kirby, 2004; Beck et al., 2011). This collective body of work was pivotal to providing a compelling historical narrative with which to engage stakeholders and justify restoration site selection (McAfee et al., 2020b).

The synthesis of historical knowledge also helped establish the case for long‐term protection of the remaining reefs, following a nomination for assessing oyster ecosystems (*O. angasi* and *S. glomerata*) as critically endangered ecological communities under Australia's *Environment Protection and Biodiversity Conservation Act 1999* (assessment under review). In overcoming historical uncertainty on their socioecological importance, ecological history helped rapidly shift shellfish reefs from relatively unknown to an ecosystem under consideration for national legislative protection and nationwide restoration.

## DEVELOPING THE BUSINESS CASE FOR INVESTMENT

Two major components of the case for restoration were foundational to building widespread interest and financial support: the socioecological history of shellfish reefs and the current socioeconomic opportunity to recover them. Shellfish reefs provide diverse goods and services of high social appeal, including enhanced fish production, water quality, shoreline protection, and ecological resilience. These ecosystem goods and services were used to indicate what has been lost and what could be gained from restoration. However, little data existed for Australian shellfish reefs in the early days of this program (2015), and no mature restorations existed to assess these services.

To build the business case for repair (priority area 2), the ecological and socioeconomic value of Australian shellfish reefs was defined by concentrating research on remnant reefs of the two most important reef‐building species (*O. angasi* and *S. glomerata*). Remnant reefs boost resident faunal communities several fold (Crawford et al., 2019; McLeod et al., 2019) and increase the resilience and adaptive capacity of these communities to climate stressors through provision of thermal refugia for resident fauna (McAfee et al., 2020a). Combined with leveraging the considerable research of the aquaculture industry on the optimal conditions for oyster growth, this body of research was used to develop reference ecosystem models for guiding restoration targets for each species (e.g., patch size, oyster density, and community assemblages) (Appendix S2). Within a matter of years, this national research effort generated a detailed knowledge base on the remnant ecological function and potential value of restored shellfish reefs (Appendix S2).

The case for restoration was largely made to policymakers on the socioeconomic opportunities for job creation and community benefit (including improved water quality and fishing opportunities). This was aided by confidence built during earlier local habitat rehabilitation initiatives (e.g., NSW's Aquatic Habitat Rehabilitation Unit) and TNC's track record of delivering logistically complex restoration projects (Schrack et al., 2012). Following early small‐scale trials and the construction of Australia's first large‐scale shellfish restoration in 2018 (Windara Reef, Appendix S1), a cost–benefit analysis identified a broad range of associated socioeconomic outcomes, such as increased fishing tourism, that could yield an estimated return on investment of two to four times (Rogers et al., 2018). Such analyses helped communicate the short‐ and long‐term socioeconomic outcomes to policymakers, with the social benefits central to the return‐on‐investment that supported the case for government funding to restore shellfish reefs. However, a comprehensive economic evaluation of the services of Australian shellfish reefs remains a knowledge gap to support future restoration work.

**FIGURE 2:**
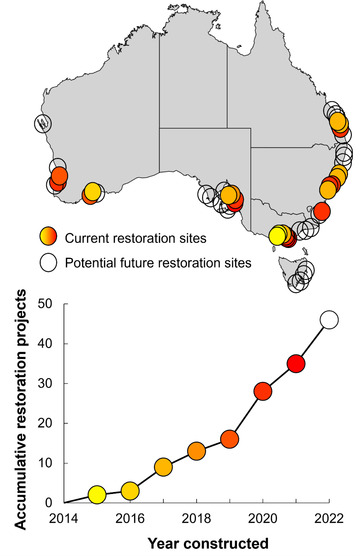
The number of shellfish reef restoration projects (list in Appendix S1) in Australia over time, from an initial restoration in 2015 to 46 projects constructed or scheduled for construction by 2022 (yellow to red gradient, earliest to the most recent restorations, respectively)

From initial meetings in 2015, support and momentum for restoration grew rapidly nationwide (Figure 2). For example, state governments initiated comprehensive programs to map remnant habitat and select sites for restoration (e.g., NSW Marine Estate Management Authority) (Appendix S2) and conducted broad community engagement campaigns to gather public opinion (McAfee et al., 2021a). Several state governments (e.g., Victoria, NSW, and South Australia) integrated shellfish reef restoration into recreational fishing enhancement programs, directly funding and even leading state‐based projects (Appendix S1). Regional recreational fishing communities provided volunteers and logistical support for restoration projects across the country and began leading increasingly ambitious restoration efforts (e.g., 19.4‐ha restoration in Moreton Bay and numerous projects across Victoria) (Appendix S1). In 2020, this momentum materialized in an AU$20 million federal government grant toward Australia's largest marine restoration program, Reef Builder: a TNC‐initiated program to restore 60 reefs nationally. By funding 13 new restoration projects in six states, this federal government investment provided economic support for coastal communities negatively affected financially by lost tourism due to COVID‐19 and the catastrophic bushfires in 2019−2020. This funding matched the ∼AU$20 million already secured for shellfish reef restoration projects in Australia through private philanthropy, corporate investment, and all tiers of government. From 2015 to 2021, ∼AU$40 million was raised for restoration, research, and community engagement. The collaboration of the multidisciplinary community working across policy, research, and community domains expedited collective efforts for management outcomes (new restoration policy), practical use (conservation and recreation), and public stewardship.

## BUILDING A NATIONAL COMMUNITY OF PRACTICE

To support nationwide research and develop skills in restoration practice (priority area 4), an informal network of coastal scientists, restoration practitioners, and policymakers came together. This network provided an important forum to generate Australia‐wide interest, expertise, and knowledge sharing on shellfish reef restoration. After the initial restoration workshop in 2015, two more national workshops followed within a year, establishing collaborative linkages and working groups dedicated to research, communication strategies, and policy pathways. Through these workshops, the Australian Shellfish Reef Restoration Network (SRRN) (https://www.shellfishrestoration.org.au/) was born, a nationwide community of practice to foster close collaboration among governments, NGOs, researchers, community groups, and industry (Appendix S1). This network continues to grow (160 members in 2021) and has expanded to include members from China and New Zealand.

Momentum for shellfish reef restoration grew from the collective effort of the SRRN. This involved clarifying the what (forgotten shellfish communities), why (they are valuable ecosystems that used to be here), how (evidence‐based restoration by experts), and who (will benefit) of shellfish reef restoration. The SRRN collectively answered questions through joint research collaboration, community workshops, international conferences, media communication, investment prospectuses to public and private funding entities, and, importantly, through published local and global guidelines for shellfish reef restoration (e.g., Fitzsimons et al., 2019) (Appendix S2).

## LEVERAGING INTERNATIONAL AND LOCAL EXPERTISE

In the absence of local examples of restoration, the case for restoration leveraged the hard‐won knowledge of half a century of shellfish reef restoration in the United States (priority area 5). In the United States, approximately 1800 restorations have recovered 4.5% of lost shellfish reef to date (Hernández et al., 2018). The social, ecological, and practical experiences of these restorations provided case studies and inspiration for Australian efforts, helping reduce some stakeholders' concerns that undertaking large‐scale restoration was too risky. Of the many lessons learned from U.S.‐based restorations, particularly important to the Australian case were three lines of evidence: that rapid reef recovery is possible (Schulte et al., 2009), providing confidence that shellfish reefs can be restored over a few years; the economic valuation of the ecological services of shellfish reef is substantial (US$5500–$99,000 ha^−1^year^−1^) (Grabowski et al., 2012); and that restored shellfish reefs boost fish productivity (zu Ermgassen et al., 2016b), which is a valuable incentive for improving Australia's fisheries. This evidence, that shellfish reefs can be rapidly restored to provide economically valuable services, was key to building early support in Australia.

Considerable local knowledge was also leveraged from Australia's oyster aquaculture industry. Beginning in the 1870s, oyster aquaculture was among colonial Australia's oldest and most important aquaculture industries (Nell, 2001), and its continued productivity built confidence that environmental conditions were suitable for restoration efforts. Decades of sustained industry research on the water and environmental conditions across which species reproduce, settle, and flourish was used for habitat suitability models for restoration. Industry techniques to overcome threats to production (e.g., disease, sedimentation, and eutrophication) now benefit restoration activities. Of note, the industry's capacity for industrial‐scale hatchery production of oysters and mussels provided confidence and boosted the operational capacity of restoration efforts. The aquaculture industry was very supportive of restoration activities, and oyster and mussel farmers were engaged through face‐to‐face talks in their farming areas to ensure no conflict occurred with lease activities (Appendix S2) (e.g., NSW Marine Estate Management Authority, 2021). Many restorations financially supported local commercial activities, such as in Victoria, where oyster seed for restoration increased the economic viability of privately run shellfish hatcheries.

## DISCUSSION

The success of a multidisciplinary approach to generate knowledge and action to restore a forgotten marine ecosystem is evident from Australia's accelerating shellfish reef restoration agenda. In just 6 years, shellfish reefs went from largely unknown ecosystems among scientists, the public, and coastal managers to being the focus of Australia's largest and most ambitious marine restoration program. This journey encompasses new research across 16 universities to date, restoration projects in all Australian states, over $40 million in blended finance, a nomination for legislative protection of the ecosystem (EPBC Act 1999), widespread public recognition and media coverage, and restoration initiatives led by NGOs, community groups, and multijurisdictional government (Appendix S1). From 2015, when Australia's first pilot reef was built, to 2021, 35 restoration projects had been constructed and 11 more were scheduled for construction across the country in 2022 (Figure 2). For the functionally extinct Australian flat oyster reefs, for which no natural reefs remain on mainland Australia, this agenda provides the opportunity to bring a marine ecosystem back from the brink of extinction.

This case study shows that success in catalyzing widespread ecosystem repair can rapidly occur when multiple parties work together with an understanding of how individual efforts contribute to the whole. From the outset, each sector worked to their expertise and collaborated across sectors to strengthen overall efforts. For example, the research community provided the critical evidence base to support recovery efforts; government agencies led ecosystem mapping, policy integration, and facilitated local community engagement; environmental and fishing conservation groups rallied local project support and volunteers; and a multinational environmental NGO with experience in large‐scale conservation was well placed to coordinate the national recovery effort. The collective agency of multiple expert parties working toward a common goal helped achieve a positive socioecological outcome that all partners could share in.

### Overcoming barriers to initiate action

Major barriers to restoration, be they political, socioeconomic, or environmental, need to be addressed if restoration is to succeed (Stewart‐Sinclair et al., 2020). The human‐centered barriers recognized in the initial workshop for this restoration agenda, such as the lack of social awareness, policy frameworks, or practical expertise in shellfish restoration (priority areas 1, 3, and 4), were overcome through two key actions. The first was the creation of the national community of practice, the SRRN. The SRRN connected experts across sectors (researchers, practitioners, government, and Indigenous communities) to build working partnerships and develop the skill‐base to deliver projects (priority area 4) and key publications (Appendices S1 & S2). This cross‐sector collaboration helped advance solutions and dialogue with policymakers, helping practitioners to navigate policy pathways to implementation. As a driving force in establishing the SRRN, TNC's international experience in restoration provided confidence, as did local expertise in shellfish aquaculture and hatchery production.

The other key action to overcome sociopolitical inertia on restoration was bringing the public and private sectors on board. A key lesson from U.S.‐based restorations was early and sustained community engagement to build support and participation during project planning and implementation (DeAngelis et al., 2020). A salient example of how this can influence restoration success comes from Florida's Mosquito Lagoon, where sustained community engagement resulted in ∼18,000 volunteers constructing almost 20,000 oyster restoration units to restore 42 degraded reefs (Schrack et al., 2012). From the outset in Australia, early community engagement focused on generating support from a key sector with many community groups: recreational fishers. This sector is politically influential in Australia, and their support is essential to improving conservation outcomes. Prior to the shellfish reef agenda, several forerunning initiatives demonstrated the value of government partnering with recreational fishers on small‐scale habitat restoration projects (Appendix S1). These grassroots projects delivered small‐scale successes that provided the evidence base, stakeholder buy‐in, and impetus for governments to support larger projects (McAfee et al., 2021b). Indeed, from grass‐roots beginnings, large‐scale recreational fisher‐led projects are now emerging. For example, OzFish Unlimited, a national recreational fishing conservation group, is restoring a 19.4 ha shellfish reef by deploying 50,000 reef units constructed by community volunteers, with similar community‐led projects underway in multiple states (Appendix S1).

**FIGURE 3:**
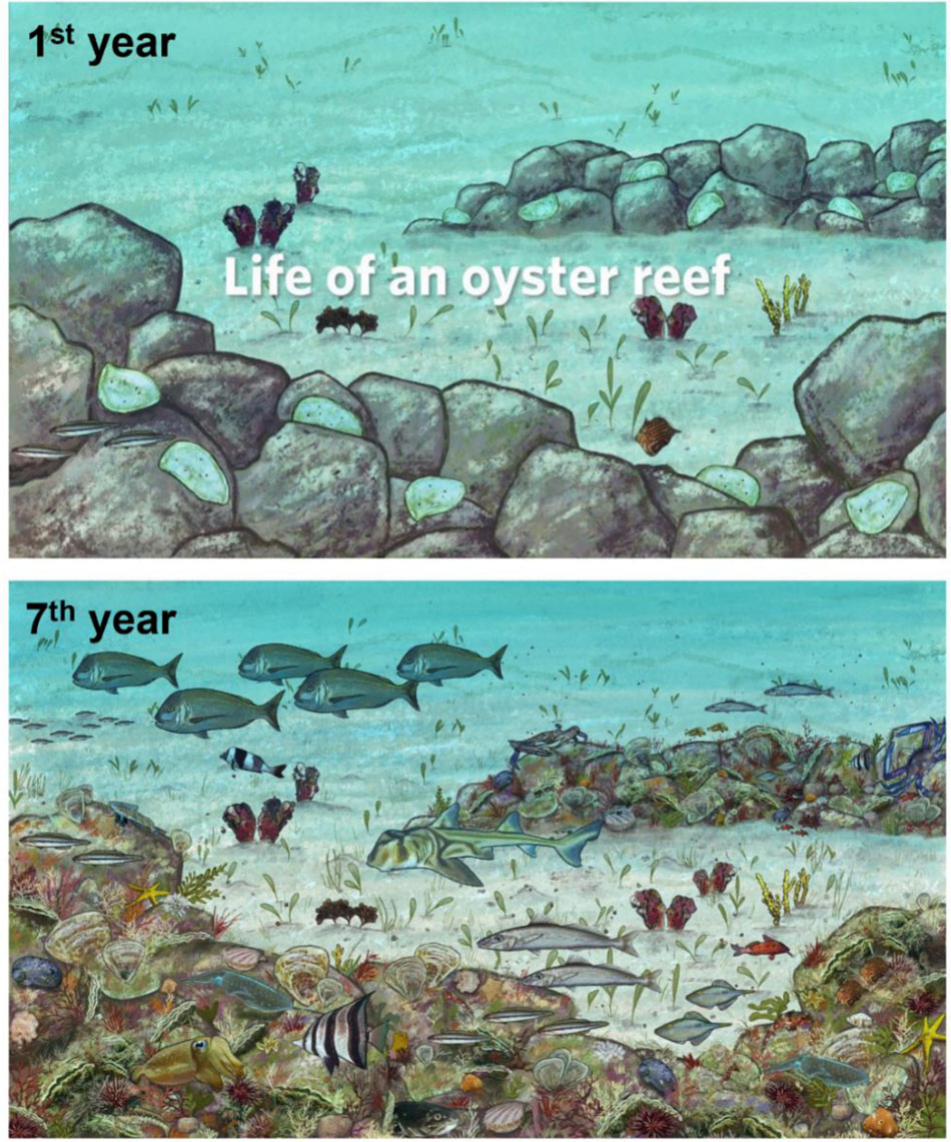
Artistic impression of how life on an oyster reef develops over the first 7 years of the reef construction. These images form part of an interactive online experience in which participants can watch and read about the incremental community change on a constructed reef (natureaustralia.org.au/what‐we‐do/our‐priorities/oceans/ocean‐stories/oyster‐reef‐habitat/)

To create awareness of shellfish reefs in the broadest audience possible, the SRRN coordinated a communication campaign that generated diverse media and educational content (i.e., website, promotional videos, and social media stories). Where project planning became formalized by government‐established working groups, various industry (fishing and tourism) and community representatives were members of advisory boards for government decision‐making (McAfee et al., 2021a). Indigenous community perspectives and knowledge was gathered through a workshop with traditional owners from across Australia and New Zealand (McLeod et al., 2018), highlighting the opportunities for comanaged restoration projects built on sustained partnerships and knowledge sharing. Finally, public participation was encouraged through numerous community forums (face‐to‐face and online). For example, the South Australian Government provided an online forum that offered the public a voice and a vote on where they wanted upcoming restorations to be located (McAfee et al., 2021a). To provide visual imagery for outreach, restoration scientists collaborated with an artist to develop a series of artistic impressions of a developing reef restoration (Figure 3) that now serves as an online educational tool (TNC's Life on an Oyster Reef [natureaustralia.org.au/what‐we‐do/our‐priorities/oceans/ocean‐stories/oyster‐reef‐habitat/]). As the scale of projects has increased, so have opportunities for community volunteering to assist with onshore preparation activities, such as shell recycling and cleaning (Branigan et al., 2020). For example, OzFish volunteers now conduct a range of activities from project fundraising and planning, to site mapping, project delivery and monitoring with guidance from local research institutions.

### Where this restoration agenda is going

The ultimate goal for this restoration agenda is to repair coastal ecosystems by restoring shellfish reefs at as many potential restoration sites within its former range (Figure 2). However, a lack of a cohesive legislative framework that works across national, state, and local jurisdictions to enable marine restoration projects remains a major hurdle for its broader uptake in Australia. A lack of appropriately prescribed terminology and specific policy or legislation also means that restoration projects must typically secure development permits intended for infrastructure projects rather than conservation (Shumway et al., 2021). This process of assessing environmental restoration as infrastructure development, which is often associated with negative environmental outcomes (e.g., construction of breakwaters), may reduce incentives for restoration due to government or corporate liability. But conversely, the process may also encourage investment in large infrastructure projects. As was recognized at the initial workshop for this restoration agenda, addressing this lack of policy cohesion remains a key objective to encourage future restoration (priority area 3). Another major challenge is restoring at ecologically meaningful scales, which is key to ensure a positive return‐on‐investment (Hernández et al., 2018). Although the practical know‐how to restore at large scales exists, delivering large‐scale projects with long‐term socioeconomic monitoring programs is constrained by public funding that is limited and distributed across priorities that constantly change. Ongoing funding sources are required that are scalable beyond current means (i.e., private sector finance).

New funding sources will likely emerge as shellfish reef restoration is increasingly incorporated into other coastal infrastructure projects. For example, opportunities exist to broaden shellfish restoration objectives and activities through alignment with marine eco‐engineering initiatives (e.g., seawalls modified to enhance biodiversity) (Bishop et al., 2022), nature‐based solutions to coastal risk reduction (Morris et al., 2021), enhancing ecosystem services (nutrient reduction and fisheries enhancement), and aquaculture programs focused on sustainable production (Jones et al., 2022). Such alignment could deliver scalable outcomes that benefit multiple partners and investors.

### Key interpretation

The rapid transition of shellfish reefs from a forgotten ecosystem to the focus of a national reef restoration program shows that transformative, socially robust conservation outcomes are possible when evidence‐based solutions act on sociopolitical opportunities. Key to this transition was the coordinated actions of a national network of collaborators working across sectors toward a common goal. Within 6 years (2015–2021), this multidisciplinary effort clarified where reefs were lost, quantified the function of remnant reefs, generated community support for reef protection, showed restoration at large scales is possible, and convinced multiple tiers of government and the private sector to invest in shellfish reef restoration as sustainable environmental policy. The program's adoption shows the political appetite exists for environmental solutions that deliver multiple socioeconomic and sustainability goals, a key part of which is aligning project goals with public and industry interests. The early social and political interest in this program suggests that society is eager to embrace policies that positively transform people's interaction with nature, and that by empowering people to contribute to and benefit from restoration these policies can change how society values nature more broadly.

## Supporting information


**Appendix S1**. List of contemporary‐era shellfish reef restoration efforts in Australia

**Appendix S2**. Published reports and practitioner guidelines to assist with knowledge‐building and restoration efforts to recover Australia's shellfish reefs
