# Supplementary material for: Turning a lost reef ecosystem into a national restoration program
Source: Conserv Biol. 2022 Aug 7;36(6):e13958. doi: 10.1111/cobi.13958 (PMC10087571; doi:10.1111/cobi.13958)
Supplement: Supplementary file 1 — Appendix S1. List of contemporary‐era shellfish reef restoration efforts in Australia [file COBI-36-0-s002.docx]

**Supporting Information**

**Appendix S1.** List of contemporary-era shellfish reef restoration efforts in Australia.

| Year built | Coast/Estuary | Reef name /location | State | Reef area | Reef base | Focal species | Lead body | Main contributing partners |
| --- | --- | --- | --- | --- | --- | --- | --- | --- |
| 2015 | Port Phillip Bay | Margaret’s Reef (Hobsons Bay) | VIC | Experimental pilot – 20 Boulder reefs (1 x 1m), 20 seeded shell (1 x 1m), and 6 mussel bed (3 x 3 m) over 0.2 ha | Limestone boulder reefs, seeded shell, live mussels | *Ostrea angasi*, *Mytilus galloprovincialis* | Victorian Fisheries Authority | Albert Park Yachting and Angling Club, The Thomas Foundation, The Nature Conservancy, Victorian Government |
| 2015 | Port Phillip Bay | Wilson Spit (Geelong Arm) | VIC | Experimental pilot – 20 Boulder reefs (1 x 1m), 20 seeded shell (1 x 1m), and 6 mussel bed (3 x 3 m) over 0.2 ha | Limestone boulder reefs, seeded shell, live mussels | *Ostrea angasi*, *Mytilus galloprovincialis* | Victorian Fisheries Authority | Albert Park Yachting and Angling Club, The Thomas Foundation, The Nature Conservancy, Victorian Government |
| 2016 | Oyster Harbour | Albany | WA | Experimental pilot – 0.0096 ha of boulder reef over 0.33 ha | Boulder reefs & live oysters | *Ostrea angasi* | The Nature Conservancy (TNC) | Recfishwest, South Coast NRM, Oyster Harbour Catchment Group, Western Australian Department of Primary Industries Fisheries, Recreational Fishing Initiative Fund, The Thomas Foundation |
| 2017 | Gulf St. Vincent | Windara Reef | SA | 15 boulder reefs over 4 ha | Limestone boulder reefs, seeded shell & live oysters | *Ostrea angasi* | South Australia’s Department of Primary Industries and Regions (PIRSA) | South Australian Department for Environment & Water, Yorke Peninsula Council, RecFish SA, TNC, Uni of Adelaide |
| 2017 | Port Phillip Bay | Margaret’s Reef (Hobsons Bay) | VIC | One boulder reef and one recycled shell reef (612 m^2^ in total) over 0.5 ha | Limestone boulder reefs, seeded shell, live mussels | *Ostrea angasi*, *Mytilus galloprovincialis* | TNC | Albert Park Yachting and Angling Club, The Thomas Foundation, Victorian Government, HSBC |
| 2017 | Port Phillip Bay | Wilson Spit (Geelong Arm) | VIC | One boulder reef and one recycled shell reef) over 0.5 ha | Limestone boulder reefs, seeded shell, live mussels | *Ostrea angasi*, *Mytilus galloprovincialis* | TNC | Albert Park Yachting and Angling Club, The Thomas Foundation, Victorian Government, HSBC |
| 2017 | Pumicestone Passage | Pacific Harbour | QLD | 16 reef units over 1 ha | Variety of boulder reefs, shell, & BESE^*^ units | *Saccostrea glomerata* | Healthy Land & Water | DigsFish Services, Traditional Owners (Joondoburri), Pumicestone Passage Fish Restocking Association, Carlo Sain, OzFish Unlimited, BCF, Uni of Sunshine Coast, Moreton Bay Regional Council |
| 2017 | Noosa Estuary | Noosa | QLD | 15 reef units throughout estuary (~11 m^2^ per reef unit) | Shell filled mesh bags | *Saccostrea glomerata* | Uni of Sunshine Coast | Noosa Biosphere Reserve Foundation, Noosa Council, Queensland Department of Agriculture and Fisheries |
| 2017 | Port Jackson | Sydney | NSW | 5 sites through Sydney area | Shell filled mesh bags | *Saccostrea glomerata* | OceanWatch Australia | Sydney Coastal Councils Group, Greater Sydney Local Land Services, Landcare NSW, NSW Rec Fishing Trust |
| 2018 | Gulf St. Vincent | Windara Reef (expansion) | SA | 144 reefs over 16 ha (20 ha total) | Limestone boulder reefs & seeded shell | *Ostrea angasi* | TNC | South Australian Department for Environment & Water, PIRSA, Yorke Peninsula Council, RecFish SA, TNC, Uni of Adelaide |
| 2018 | Port River (Adelaide) | Port Adelaide | SA | Not reported | Shell filled mesh bags & terracotta tiles | *Ostrea angasi* | Estuary Care Foundation | Green Adelaide NRM, local oyster farmers, Marine Life Society SA, TNC, Uni of Adelaide |
| 2018 | Pumicestone Passage | Pacific Harbour | QLD | 0.006 ha of constructed reef | Shell & concrete blocks | *Saccostrea glomerata* | Healthy Land & Water | OzFish Unlimited, Australian Government, DigFish Services, BCF, Traditional Owners (Ngunda-Joondooburri Trust, Kabi Kabi First Nation), Uni of Sunshine Coast, Pumicestone Passage Fish Restocking Association, Moreton Bay Regional Council, Sunfish, Carlo Sain, Unitywater, Queensland Government |
| 2018 | Brisbane Water Estuary | Brisbane Water | NSW | 0.003 ha of constructed reef | Shell filled mesh bags | *Saccostrea glomerata* | OceanWatch Australia | Sydney Coastal Councils Group, Greater Sydney Local Land Services, Landcare NSW, NSW Rec Fishing Trust |
| 2019 | Swan-Canning Estuary | Perth | WA | 16 (7 x 5m) pilot reefs at four locations throughout lower Swan-Canning Estuary | Limestone boulder reefs & live mussels | *Mytilus galloprovincialis* | TNC | Minderoo Foundation, WA Department of Biodiversity, Conservation and Attractions |
| 2019 | Port Phillip | Wilson Spit (expansion) | VIC | Two recycled shell reefs over 0.5 ha (total 1 ha) | Limestone boulder reefs, seeded shell, live mussels | *Ostrea angasi*, *Mytilus galloprovincialis* | TNC | Albert Park Yachting and Angling Club, The Thomas Foundation, Victorian Government, HSBC |
| 2019 | Oyster Harbour | Albany | WA | 2 boulder reefs over 1 ha | Boulder reefs & live oysters | *Ostrea angasi* | TNC | Government of Western Australia, Recfishwest, Great Southern Development Commission, South Coast NRM Inc., the Uni of Western Australia, Albany Shellfish Hatchery |
| 2020 | Glenelg | Glenelg Reef | SA | 14 boulder reefs over 2 ha | Limestone boulder reefs & seeded shell | *Ostrea angasi* | TNC | City of Holdfast Bay, SA Department for Environment & Water |
| 2020 | Port River (Adelaide) | Port Adelaide | SA | Not reported | Shell filled mesh bags | *Ostrea angasi* | Estuary Care Foundation | Green Adelaide NRM, Portside Christian College, OzFish Unlimited |
| 2020 | Moreton Bay | Port of Brisbane | QLD | Expanding to 19.4 ha over 5 years | Triangular gabions filled with seeded and unseeded recycled shell | *Saccostrea glomerata* | OzFish Unlimited | Healthy Land and Water, Australian Government, Queensland Government, BCF, The Moreton Bay Foundation, Tackle Tactics, Iona College, various Queensland Rotary clubs, Turner Family Foundation, Port of Brisbane, Signato Foundation, Griffith Uni, Uni of Queensland, Uni of Sunshine Coast |
| 2020 | Port Phillip Bay | Hobsons Bay  (expansion) | VIC | 2 Boulder reefs over 0.5 ha (total 1 ha) | Limestone boulder reefs, seeded shell, live mussels | *Ostrea angasi*, *Mytilus galloprovincialis* | TNC | Albert Park Yachting and Angling Club, Victorian Fisheries Authority, Victorian Government |
| 2020 | Port Phillip Bay | Wilsons Spit  (expansion) | VIC | 5 recycled shell and boulder reefs over 1.5 ha (total 2.5 ha) | Limestone boulder reefs, seeded shell, live mussels | *Ostrea angasi*, *Mytilus galloprovincialis* | TNC | Albert Park Yachting and Angling Club, Victorian Fisheries Authority, Victorian Government |
| 2020 | Port Phillip Bay | Dromana | VIC | 5 boulder reefs over 1 ha | Limestone boulder reefs, seeded shell, live mussels | *Ostrea angasi*, *Mytilus galloprovincialis* | TNC | Ross Trust, Victorian Fisheries Authority, Victorian Government |
| 2020 | Port Phillip Bay | 9ft Bank Shellfish Reef | VIC | 0.016 ha of constructed reef | Recycled shell reef | *Ostrea angasi* | TNC | Lord Mayor’s Charitable Foundation, Victorian Fisheries Authority, Victorian Government |
| 2020 | Port Phillip Bay | Merv’s Reef (Corio Bay) | VIC | 5 boulder reefs | Basalt and limestone boulder reefs, seeded shell and live mussels | *Ostrea angasi*, *Mytilus galloprovincialis* | Victorian Fisheries Authority | Victorian Government, VRFish, City of Greater Geelong |
| 2020 | Port Phillip Bay | Wilson’s Reef (Geelong Arm) | VIC | 6 boulder reefs | Basalt and limestone boulder reefs, seeded shell and live mussels | *Ostrea angasi*, *Mytilus galloprovincialis* | Victorian Fisheries Authority | Victorian Government, VRFish, City of Greater Geelong |
| 2020 | Port Phillip Bay | Moolap Reef (Geelong Outer Harbour) | VIC | 6 boulder reefs | Basalt and limestone boulder reefs, seeded shell and live mussels | *Ostrea angasi*, *Mytilus galloprovincialis* | Victorian Fisheries Authority | Victorian Government, VRFish, City of Greater Geelong |
| 2020 | Karuah River & Myall River | Port Stephens | NSW | 1 ha of constructed reef at 2 sites | Andesite boulder reefs & shell | *Saccostrea glomerata* | New South Wales Department of Primary Industries (NSW DPI) | Local oyster farmers |
| 2020 | Bermagui River, Port Hacking, Port Stephens | NSW | NSW | 6 sites | BESE units* | *Saccostrea glomerata* | Macquarie Uni | New South Wales Environmental Trust, NSW DPI Fisheries |
| 2021 | Karuah River & Myall River | Port Stephens  (2 sites expanded) | NSW | 1.5 ha of constructed reef at 2 sites (2.5 ha total) | Andesite boulder reefs & shell | *Saccostrea glomerata* | NSW DPI | Australian Government, TNC, local oyster farmers |
| 2021 | Hastings River | Port Macquarie | NSW | Not reported | Boulder reefs | *Saccostrea glomerata* | OceanWatch Australia | North Coast Local Land Services, Australian Government, NSW DPI Fisheries, local oyster farmers |
| 2021 | Port Phillip Bay | Hobsons Bay  (expansion) | VIC | 10 Boulder reefs over 1.5 ha (2.5 ha total) | Limestone boulder reefs, seeded shell, live mussels | *Ostrea angasi*, *Mytilus galloprovincialis* | TNC | Albert Park Yachting and Angling Club, Australian Government |
| 2021 | Port Phillip Bay | Wilsons Spit  (expansion) | VIC | 13 reefs over 2.5 ha (5 ha total) | Limestone boulder reefs, seeded shell, live mussels | *Ostrea angasi*, *Mytilus galloprovincialis* | TNC | Albert Park Yachting and Angling Club, Australian Government |
| 2021 | Port Phillip Bay | Dromana (expansion) | VIC | 7 boulder reefs over 1 ha (total 2 ha) | Limestone boulder reefs, seeded shell, live mussels | *Ostrea angasi*, *Mytilus galloprovincialis* | TNC | Albert Park Yachting and Angling Club, Australian Government |
| 2021 | Glenelg | Glenelg Reef (expansion) | SA | 7 boulder reefs over 3 ha (5 ha total) | Limestone boulder reefs & seeded shell | *Ostrea angasi* | TNC | City of Holdfast Bay South Australian Department for Environment & Water, Australian Government, Uni of Adelaide |
| 2021 | O’Sullivan Beach | Onkaparinga | SA | 22 reefs over 5 ha | Limestone boulder reefs & seeded shell | *Ostrea angasi* | TNC | City of Onkaparinga, South Australian Department for Environment & Water, Australian Government, Uni of Adelaide |
| 2022 | Noosa Estuary | Noosa | QLD | To be constructed | Boulder reefs | *Saccostrea glomerata* | TNC | Noosa Shire Council and the Noosa community, The Thomas Foundation, Australian Marine Conservation Society, Australian Government |
| 2022 | Wagonga Estuary | Wagonga | NSW | To be constructed | Boulder reefs & seeded shell | *Saccostrea glomerata, Ostrea angasi* | NSW DPI | TNC, Australian Government, Eurobodalla Shire Council |
| 2022 | Botany Bay | Sydney | NSW | To be constructed | Boulder reefs (seeded and unseeded) | *Saccostrea glomerata, Ostrea angasi* | TNC | Greater Sydney Local Land Services, Australian Government, NSW DPI Fisheries, Recreational Fishers |
| 2022 | Kingscote | Kangaroo Island | SA | To be constructed | Various reef materials | *Ostrea angasi* | Kangaroo Island Landscape Board | TNC, South Australian Department for Environment & Water |
| 2022 | America River | Kangaroo Island | SA | To be constructed | Boulder reefs | *Ostrea angasi* | TNC | Australian Government, SA Department for Environment & Water |
| 2022 | D’Entrecasteaux Channel & Derwent Estuary | South-east Tasmania | TAS | To be constructed | Boulder reefs | *Ostrea angasi* | NRM South | TNC, Australian Government |
| 2022 | Swan-Canning Estuary | Perth | WA | To be constructed | Boulder reefs | *Mytilus galloprovincialis* | TNC | Minderoo Foundation, Western Australian Department of Biodiversity, Conservation and Attractions, Australian Government |
| 2022 | Peel-Harvey Estuary | Mandurah | WA | To be constructed | Boulder reefs | *Mytilus galloprovincialis* | TNC | Alcoa Foundation, Australian Government |
| 2022 | Oyster Harbour (Expansion) | Albany | WA | To be constructed | Boulder reefs | *Ostrea angasi* | TNC | Government of Western Australia, Recfishwest, Great Southern Development Commission, South Coast NRM Inc., Uni of Western Australia, Albany Shellfish Hatchery |
| 2022 | Port Phillip Bay | Indented Head | VIC | To be constructed | Boulder reefs and recycled shell | *Ostrea angasi, Mytilus galloprovincialis* | OzFish Unlimited | Australian Government, Corangamite CMA, VRFish |
| 2022 | Port Phillip Bay | Mornington/Mt Eliza | VIC | To be constructed | Boulder reefs and recycled shell | *Ostrea angasi, Mytilus galloprovincialis* | OzFish Unlimited | Australian Government, Melbourne Water, VRFish |

*BESE units – Biodegradable Ecosystem Engineering units (BESE Products 2020) are interlocking sheets of biodegradable mesh that provide a settlement matrix for invertebrates.

*Notes on contributors to shellfish restoration in Australia*:

- As contributors to Australia’s Shellfish Reef Restoration Network (SRRN), diverse government bodies and public officers help facilitate discussions with policymakers and improve policy pathways to enable these restoration efforts, including (but not limited to):

New South Wales (NSW): Department of Primary Industries, Department of Planning, Industry and Environment; Queensland (QLD): Department of Agriculture and Fisheries; South Australia (SA): Department for Environment and Water, Department of Primary Industries and Regions South Australia; Victoria (VIC): Victorian Fisheries Authority; Western Australia (WA): Department of Primary Industries and Regional Development, Department of Biodiversity, Conservation and Attractions

- As the major research providers of the SRRN, at least 16 universities across the country have contributed research during the planning, implementation, and monitoring stage of these restoration projects, including:

University of Adelaide, Flinders University, Griffith University, James Cook University, Macquarie University, University of Melbourne, Murdoch University, University of Newcastle, University of New South Wales, University of Queensland, Southern Cross University, University of the Sunshine Coast, University of Sydney, University of Tasmania, University of Technology Sydney, University of Western Australia

- Many recreational fishing bodies, conservation groups, and oyster and mussel farmers have contributed to the SRRN and individual projects, including:

Albert Park Yachting & Angling Club (VIC), Estuary Care Foundation (SA), OzFish Unlimited (national), Oyster Harbour Catchment Group (WA), Pacific Estate Oysters (SA), state-based recreational fishing bodies (e.g., Recfishwest, RecFish SA, VRFish)

*Notable forerunning initiatives:*

- State Government Recreational Fishing Trusts

Beginning 2001, the NSW Department of Primary Industries and the Victorian Fisheries Authority established Recreational Fishing Trusts with funds generated by annual recreational fishing licence fees (e.g., generating AUD $16.8 million from ~474,000 fishing licences in NSW alone in 2021, and approximately AUD $ 9 million in Victoria from over 250,000 licences). Most of the money raised by these Trusts is spent on the rehabilitation and protection of fish habitat, research on fish ecology, fish stocking, improvement to access and facilities, education and promoting compliance with fishing regulations in each state. These Trusts provide grant funding to recreational fishing clubs, research groups, and communities groups to support typically small-scale habitat rehabilitation and restoration work (e.g., NSW’s Habitat Action Grants). To date, hundreds of community and recreational fisher-led projects have improved riverine and coastal habitat across each State. Notably, in 2015, the Victorian Recreational Fishing Grants Program granted the Albert Park Yachting & Angling Club $147,000 to create the first shellfish restoration reefs in Port Phillip Bay.

- National Estuaries Network, Australian Government

Established in 2000, the National Estuaries Network is a national forum that was created to link coastal managers across State borders with estuarine scientists to improve the management and conservation of estuaries throughout Australia. Activities included the create of a publicly accessible national database of Australia’s estuaries and coastal waterways (OzCoasts), a national collaboration by more than 100 coastal scientists from diverse government agencies and universities. Available here: [https://ozcoasts.org.au/nen/](about:blank)
