# Supplementary material for: Turning a lost reef ecosystem into a national restoration program
Source: Conserv Biol. 2022 Aug 7;36(6):e13958. doi: 10.1111/cobi.13958 (PMC10087571; doi:10.1111/cobi.13958)
Supplement: Supplementary file 2 — Appendix S2. Published reports and practitioner guidelines to assist with knowledge‐building and restoration efforts to recover Australia's shellfish reefs [file COBI-36-0-s001.docx]

**Supporting Information**

**Appendix S2.** Published reports and practitioner guidelines to assist with knowledge building and restoration efforts to recover Australia’s shellfish reefs

*Reports on the current state and ecological role of Australian shellfish reefs:*

Gillies, C. L., Creighton, C., & McLeod, I. M. (eds) (2015). **Shellfish reef habitats: a synopsis to underpin the repair and conservation of Australia’s environmentally, socially and economically important bays and estuaries**. Report to the National Environmental Science Programme, Marine Biodiversity Hub. Centre for Tropical Water and Aquatic Ecosystem Research (TropWATER) Publication, James Cook University, Townsville, 68 pp. Accessible at: <https://www.shellfishrestoration.org.au/resources/>

McLeod, I. M., & Böstrom-Einarsson, L. (2019). **Shellfish Reefs**; chapter 4 in: **The role of restoration in conserving matters of national environmental significance in marine and coastal environments**. Report to the National Environmental Science Programme, Marine Biodiversity Hub. Accessible at: <https://www.nespmarine.edu.au/project/project-e5-role-restoration-conserving-matters-national-environmental-significance>

*Publications describing the historical loss of shellfish reefs at local and national scales:*

Hamer, P., Pearce, B., Winstanley, R. (2013). **Towards reconstruction of the lost shellfish reefs of Port Phillip Bay**. Recreational Fishing Grants Program Research Report (project SG/117). Department of Environment and Primary Industries, Victoria Government.

Diggles, B. K. (2013**). Historical epidemiology indicates water quality decline drives loss of oyster (*Saccostrea glomerata*) reefs in Moreton Bay, Australia**. New Zealand Journal of Marine and Freshwater Research, 47(4), 561-581.

Alleway, H. K., & Connell, S. D. (2015). **Loss of an ecological baseline through the eradication of oyster reefs from coastal ecosystems and human memory**. Conservation Biology, 29(3), 795-804.

Ford, J. R., & Hamer, P. (2016). **The forgotten shellfish reefs of coastal Victoria: documenting the loss of a marine ecosystem over 200 years since European settlement**. Proceedings of the Royal Society of Victoria, 128(1), 87-105.

Gillies, C. L., McLeod, I. M., Alleway, H. K., et al. (2018). **Australian shellfish ecosystems: Past distribution, current status and future direction**. PLoS One, 13(2), e0190914.

Gillies, C. L., Castine, S. A., Alleway, H. K., et al. (2020). **Conservation status of the oyster reef ecosystem of southern and eastern Australia.** Global Ecology and Conservation, 22, e00988.

Thurstan, R. H., Diggles, B. K., Gillies, C. L., et al. (2020). **Charting two centuries of transformation in a coastal social-ecological system: A mixed methods approach**. Global Environmental Change, 61, 102058.

Cook, P. A., Warnock, B., Gillies, C. L., & Hams, A. B. (2021). **Historical abundance and distribution of the native flat oyster (*Ostrea angasi*) in estuaries of the Great Southern region of Western Australia help to prioritise potential sites for contemporary oyster reef restoration**. Marine and Freshwater Research, 73(1), 48-56.

*Guidelines for shellfish reef restoration (local and global guidelines):*

Fitzsimons, J., Branigan, S., Brumbaugh, R.D., McDonald, T. and zu Ermgassen, P.S.E. (eds) (2019). **Restoration Guidelines for Shellfish Reefs**. The Nature Conservancy, Arlington VA, USA. Accessible at: <https://www.shellfishrestoration.org.au/resources/>

Fitzsimons, J. A., Branigan, S., Gillies, C. L., Brumbaugh, R. L., Cheng, J., DeAnglis, B. M., Geselbracht, L., Hancock, B., Jeffs, A., McDonald, T., McLeod, I. M., Pogoda, B., Theuerkauf, S. J., Thomas, M., Westby, S., zu Ermgassen, P. S. E. (2020). **Restoring shellfish reefs: Global guidelines for practitioners and scientists**. Conservation Science and Practice, 2(6), e198.

NSW Marine Estate Management Authority (2021). **NSW Shellfish Reef Restoration Project Planning and Implementation Guidelines**. NSW Department of Primary Industries. Accessible at: <https://www.marine.nsw.gov.au/__data/assets/pdf_file/0003/1322526/16741-Shellfish-Reef-Guidelines-A-1.pdf>

*Reference ecosystem models for Australian flat oysters and Sydney rock oysters:*

Gillies, C. L., Crawford, C., & Hancock, B. (2017). **Restoring Angasi oyster reefs: What is the endpoint ecosystem we are aiming for and how do we get there?** Ecological Management & Restoration, 18(3), 214-222.

McAfee, D., McLeod, I. M., Boström‐Einarsson, L., & Gillies, C. L. (2020). **The value and opportunity of restoring Australia's lost rock oyster reefs**. Restoration Ecology, 28(2), 304-314.

*Benefit-cost analysis for shellfish reef restoration (Windara Reef case-study):*

Rogers, A. A., Nedosyko, A., McLeod, I. M., Gillies, C., & Burton, M. P. (2018). **Benefit-cost analysis of the Windara shellfish reef restoration project**. Project B1: Road testing decision support tools via case study applications. Report to the National Environmental Science Program, Marine Biodiversity Hub. The University of Western Australia. Accessible at: <https://www.nespmarine.edu.au/document/benefit-cost-analysis-windara-shellfish-reef-restoration-project>

*Australian guide to nature-based coastal defence:*

Morris, R. L., Bishop, M. J., Boon, P., Browne, N. K., Carley, J. T., Fest, B. J., Fraser, M. W., Ghisalberti, M., Kendrick, G. A., Konlechner, T. M., Lovelock, C. E., Lowe, R. J., Rogers, A. A., Simpson, V., Strain, E. M. A., Van Rooijen, A. A., Waters, E., & Swearer, S. E. (2021). **The Australian Guide to Nature-Based Methods for Reducing Risk from Coastal Hazards**. Report (No. 26. ) to the National Environmental Science Program, Earth Systems and Climate Change Hub, Australia. Accessible at: <https://nespclimate.com.au/australian-guidelines-for-the-implementation-of-nature-based-methods-for-coastal-hazard-risk-reductio/>
